# 

**Published:** 1839-08

**Authors:** 


### THE MARYLAND MEDICAL AND SURGICAL JOURNAL.

We have received the prospectus of a periodical to be published quarterly, in Baltimore, by the *Medical and Chirurgical Faculty of Maryland*, under the above title; and to the editorship of which, the following gentlemen were elected by the faculty at its last annual Meeting: G. C. M. Roberts, M. D., Nathaniel Potter, M. D., James H. Miller, M. D. Robert A. Durkee, M. D., J. R. W. Dunbar, M. D., and Samuel George Baker, M. D.

That the work will be conducted with ability, the names of the gentlemen, under whose superintendence it has been placed, are a sufficient guaranty; and from the favorable auspices under which it is thus to be brought out, we doubt not that it will be well sustained. That a publication of this kind is needed in Baltimore, is generally acknowledged.—No city of the same size should be without one, and notwithstanding the many similar ones which we already have in the country, there is room for more. Diseases are constantly changing and assuming different forms, and improvements are daily being made both in medicine and surgery.—With these every practitioner should be made acquainted, and there is no way by which they can be so readily communicated as through the medium of works of this kind. Each number of the "Maryland Medical and Surgical Journal," is to contain at least 128 pages, and is to be published at the low price of $2,50 in advance, or three dollars if deferred. The first number, we understand, is to be brought out sometime during the present month, July. H.

---

It is a fact perhaps not generally known to the profession, that the father of Francis Joseph Talma, the greatest of French Actors, was a dentist, resident for some years in England. Talma himself followed the profession of his father for eighteen months in Paris, but left it for the stage in 1787. He was highly esteemed both as an actor and a man, by Napoleon.

---

TO SUBSCRIBERS.—During the present year, the Publishing Committee may perhaps deem it expedient to deviate from the general rule of giving monthly numbers. It has been recommended that the Journal be published quarterly, which suggestion may at some future period be adopted; and for the present it may not always be convenient on account of arduous professional and other duties, to have the numbers ready for delivery exactly on the first of every month; but subscribers may rely on having the full amount of matter promised in the prospectus although more than twelve months may be required to complete the contract.

### ADDRESS TO THE PROFESSION.

---

Of the utility of a periodical, such, as it is proposed to make this, especially to the dental profession, none can doubt. Little need, therefore, be said by way of eulogy. Its very name will, we feel assured, at once commend it to the patronage of every respectable dentist; for in none of the departments of the great medical art, is a work of this kind so much needed as in this. By reason of the scarcity of works on dentistry, many of its practitioners are unable to avail themselves of the experience of others; and in the exercise of their profession are often compelled to depend solely on the suggestions of their own imaginations. The literature of all the other departments of medicine, although already occupying the pages of vast numbers of large volumes, is still being daily increased and enriched by periodical publications. By this means, the practitioners of medicine and surgery are kept constantly advised of all the improvements in their respective branches.

But not so with dentists, for even the works of many of the most celebrated English Authors in dental science, are either inaccessible or out of print; and many of the best works on dentistry, being in foreign languages, are unavailable to the great majority of the profession in America. It is to supply this deficiency, and to furnish to practitioners the latest improvements in the art, that the publication of "*The American Journal of Dental Science*" has been undertaken. That the design will be appreciated by the profession, we entertain not the slightest doubt. Of the importance of constantly adding to our stock of knowledge, by keeping pace with the improvements of the art, we are all well aware. This we owe to ourselves—to the community upon whom we are dependent for support, and to the profession. Policy, and self-respect, if nothing else, require it; and to the attainment of this object, the present publication will be found highly conducive; for upon its pages will be spread the experience of the past, and the improvements of the present.

In making this appeal to the profession, we do it in a spirit of confidence, which, from the encouragement we have already received, we feel will not be misplaced. Many have already subscribed, some for several copies; and all that is wanting to insure its permanent success, is, for the profession generally, throughout the United States, to extend to it a helping hand. It is, therefore, necessary, that those who intend to subscribe to it, should send on their names immediately, with the amount of subscription.

We would also, in this undertaking, respectfully solicit the aid of the medical public. To physicians residing in places where the services of the dentist cannot always be procured, the American Journal of Dental Science will be of great utility; for, by acquainting them with the diseases of the teeth, and the means necessary for their cure, it will often enable them to treat successfully such diseases of other parts of the body as have their origin in an unhealthy condition which, otherwise, they would often be unable to accomplish. The diseases of these organs, the teeth, have, hitherto, attracted too little attention from medical men. They have not engaged as much of their attention as the importance calls for; but the want of information in medical works, on this subject, may, by the present publication, be supplied.

Nor will the usefulness of this work be altogether confined to the dental and medical practitioner: it will be found interesting and valuable to all who regard the health and preservation of their teeth as of importance. The means necessary to the attainment of this object will be carefully pointed out, so that by their proper observance, many of the diseases to which these invaluable organs are liable, may be avoided, and when the services of a dentist become necessary those skilled in the profession will be employed.

TERMS.—The AMERICAN JOURNAL OF DENTAL SCIENCE will be published monthly, on good paper, with new type, each number containing 48 octavo pages, stitched in a printed cover, making at the end of the year a volume of 576, or two volumes of 288 pages each, at $3 per annum, payable in advance. Two copies will be sent for $5.

All communications must be post paid, and directed to Solyman Brown, Dentist, 17 Park Place, New York, Secretary of the Publishing Committee.

Cash remittances should be directed to Mr. Jahial Parmly, Treasurer, No. 11 Park Place, New-York.

Editors of newspapers, to whom the first number of this Journal was sent, will, by copying the foregoing prospectus, have it continued for 12 months gratis, if they forward a copy of the paper containing the advertisement to the Secretary of the Publishing Committee.

---

Inasmuch as a sufficient number of Subscribers for single copies, or for duplicates of this Journal, did not proffer their support in time to issue a second number during the month of July, the following persons have volunteered their aid by subscribing for twenty copies or more.

If any individuals are disposed to add their names to this catalogue with a view to aid in the circulation of the Journal, they are requested to express their intention to the Publishing Committee, before the appearance of the next number.

Subscribers for less than 20 copies will not be inserted on this page.

### SPECIAL PATRONS:

| | | | |
|---|---|---|---|
| ELEAZAR PARMLY, *Dentist, N. York,* - - - | 40 | copies, | $100 |
| CHAPIN A. HARRIS, M. D. *Dentist, Baltimore* - | 40 | " | 100 |
| GEORGE KEARSING, *Goldbeater, N. York,* - - | 20 | " | 50 |
| E. NOYES, *Dentist, Baltimore,* - - - - - - - | 20 | " | 50 |
| LEVI S. PARMLY, *Dentist, New Orleans,* - - - | 20 | " | 50 |
| W. N. McKENNEY, *Dentist, Fredericksburg, Va.* - | 20 | " | 50 |
| JOHN BURDELL, *Dentist, New-York,* - - - | 20 | " | 50 |
| WALKER & JONES, *Goldbeaters, New-York,* - | 20 | " | 50 |
| SAM'L W. STOCKTON, *Dentist, Phila.* - - - | 20 | " | 50 |
| JAHIAL PARMLY, *Dentist, New-York,* - - - | 20 | " | 50 |
| E. BAKER, *Dentist, New-York,* - - - - - - | 20 | " | 50 |
| A. WOODRUFF BROWN, *Dentist, New-York,* - | 20 | " | 50 |
| EDW'D MAYNARD, *Dentist, Washington City,* - | 20 | " | 50 |
| J. A. FRAETAS, *Printer, New-York,* - - - - | 20 | " | 50 |

---

### TO CONTRIBUTORS.

The Publishing Committee have received several Communications for the pages of the Journal, which, for want of room, they are compelled to reserve for the next number. Well written articles will be thankfully received from all parts of the country, especially if they discuss subjects of practical utility to the dental student.

AGENTS FOR THE

AMERICAN JOURNAL OF DENTAL SCIENCE.

Doct. W. N. McKenney, *Fredericksburg, Va.*

F. B. Chewning, *Richmond, Va.*

A. Pierce, *Sussex Court House, Va.*

Chas. S. Pleasants, *Painesville, Ohio.*

David Walker & Asahel Jones, 174 *Fulton Street, New York.*

Edward Taylor, *Rochester.*

O. P. Laird, M. D., *Oneida Castle, New York.*

John D. Chevalier, 135 *Fulton Street, New-York.*

F. M. Boykin, M. D. *Smithfield, Va.*

G. McDonald, M. D. *Macon, Geo.*

E. Maynard, M. D. *Washington City.*

McAllister & McNaughton, *Albany.*

M. R. Bridges, *Brooklyn.*

Blanding & Avery, *Columbia, S. C.*

Eleazer Gidney, *England.*

Sam'l Wheeler, M. D. *Murfreesboro, N. C.*

W. H. H. Davis, M. D. *Cassville, Oneida Co. New-York.*

